# Diagnosis and Management of Hepatobiliary Complications in Autosomal Recessive Polycystic Kidney Disease

**DOI:** 10.3389/fped.2017.00124

**Published:** 2017-05-29

**Authors:** Andrew Wehrman, Alyssa Kriegermeier, Jessica Wen

**Affiliations:** ^1^The Children’s Hospital of Philadelphia, Philadelphia, PA, United States

**Keywords:** autosomal recessive polycystic kidney disease, congenital hepatic fibrosis, cholangitis, portal hypertension, esophageal varices

## Abstract

Autosomal recessive polycystic kidney disease (ARPKD) is a congenital hepatorenal fibrocystic disease. The hepatic manifestations of ARPKD can range from asymptomatic to portal hypertension and massively dilated biliary system that results in liver transplantation. Hepatic complications of ARPKD typically present with signs of portal hypertension (splenomegaly and thrombocytopenia) or cholangitis. Liver disease in ARPKD does not always correlate with severity of renal disease. Management of ARPKD-related liver disease is largely treating specific symptoms, such as antibiotics for cholangitis or endoscopic treatment for variceal bleeding. If complications cannot be managed medically, liver transplantation may be indicated. This mini-review will discuss the clinical manifestations and management of children with ARPKD liver disease.

## Introduction

Autosomal recessive polycystic kidney disease (ARPKD) is a fibrocystic disease of the kidney and liver that often presents in childhood with renal disease. The most severe form is associated with *in utero* findings of oligohydramnios, which can lead to pulmonary hypoplasia and result in mortality in the neonatal period. The incidence of ARPKD is approximately 1:16,000 live births and is caused by a mutation in PKHD1 gene, which encodes the protein fibrocystin (1). Clinically apparent hepatic involvement occurs in approximately 45% of ARPKD cases and is manifested by ductal plate malformations including cystic biliary disease and congenital hepatic fibrosis (CHF) (1). Patients with significant hepatic disease can present with portal hypertension, manifested by thrombocytopenia, splenomegaly, and esophageal variceal hemorrhage. Biliary duct ectasia also predisposes these patients to recurrent cholangitis. There is no specific cure for ARPKD/CHF, and management of hepatic manifestations is largely supportive. This paper aims to review the clinical presentation, diagnosis, and treatment of hepatobiliary disease in patients with ARPKD.

## Ductal Plate Malformations

Hepatic manifestations of ARPKD are caused by alterations in the normal development of the biliary ducts due to defects in the primary cilia of cholangiocytes. Broadly, developmental disorders of the intrahepatic bile ducts, including those associated with ARPKD, namely Caroli disease/syndrome and CHF, are due to ductal plate malformations. The ductal plate is an embryologic structure, which initially consists of a sheath of cells surrounding the hilar portal vein branches. During development, the ductal plate reorganizes and progresses peripherally leading to the formation of bile ducts and ductules. In normal development, the remaining regions of the ductal plate regress. When the ductal plate fails to regress, these embryologic structures persist, with the potential to become massively dilated and manifest as cystic lesions that are in connection with the biliary tree (2, 3).

Ductal plate abnormalities can manifest in several ways histologically and radiographically including CHF, Caroli syndrome, and Caroli disease. Caroli disease refers to isolated congenital dilation of intrahepatic biliary ducts and is an uncommon presentation in ARPKD (4). Caroli syndrome refers to cases where in addition to dilation of the intrahepatic biliary tree, there is CHF due to malformations of the small bile ductules leading to fibrosis. Caroli syndrome and CHF are seen in a variety of conditions related to primary ciliopathies, including ARPKD (2).

## Clinical Features and Diagnosis of Liver Disease in ARPKD

Hepatic manifestations of ARPKD in children often present with signs of portal hypertension, which can include splenomegaly, thrombocytopenia, ascites, and recurrent gastrointestinal bleeding (melena or hematemesis). Liver enzymes and bilirubin levels may be normal or minimally elevated in CHF. Elevations in GGT can be seen due to cystic dilation of the biliary tree. Liver synthetic function is typically intact; however, coagulation studies should be monitored to evaluate for vitamin K malabsorption due to cholestasis (5). Liver biopsy is not usually indicated if there is genetic diagnosis. However, biopsy may occasionally be considered as part of the evaluation for CHF if diagnosis is not clear or bacterial culture from bile is required in those with persistent bacterial cholangitis not responsive to empiric antibiotic treatment. Imaging studies such as ultrasound (US) and MR cholangiogram may be needed for establishing diagnosis or for monitoring (discussed below). The severity of liver disease is not dependent on the severity of kidney disease. Liver disease has the potential to progress independent of kidney disease, and vice versa.

A significant morbidity and mortality of ARPKD is due to esophageal varices and bleeding. Liver disease associated with ARPKD has the unique features of significant portal fibrosis without inflammatory infiltrate unless the disease has been complicated by recurrent cholangitis. Therefore, most patients have well preserved hepatocyte synthetic function but often can present with massive splenomegaly and esophageal variceal bleeding.

Given the large cystic dilation of the biliary tree, patients often have bile stasis, which puts them at risk for cholangitis and sepsis as well as intra or extra-hepatic cholelithiasis. Recurrent cholangitis may hasten progression to cirrhosis and failure to eradicate the infection may be an indication for liver transplantation. Additionally, older patients with liver involvement have an increased risk of cholangiocarcinoma (5–10%) (6). Carbohydrate antigen 19-9 and carcinoembryonic antigen can be used to screen for cholangiocarcinoma in young adults, although there is no screening guideline recommended for this special population.

## Imaging Findings in ARPKD

As discussed previously, the most common hepatic manifestation of ARPKD is Caroli syndrome. Hepatic abnormalities in CHF and Caroli syndrome can be readily seen on imaging modalities. In patients with ARPKD-associated liver disease, the liver is often asymmetrical with an enlarged left lateral segment (7). The hallmark biliary lesions seen on imaging are large, ectatic bile ducts that communicate with the biliary tree. Liver fibrosis can be seen described on some imaging modalities, but extent of fibrosis is difficult to measure on imaging alone. Signs of portal hypertension, such as splenomegaly, can be seen on abdominal US. Reversal of flow in the portal vein can also be indicative of portal hypertension, but this is not required for diagnosis. Transient elastography has been studied in ARPKD (8), but this has not been widely used.

**Figure 1:**
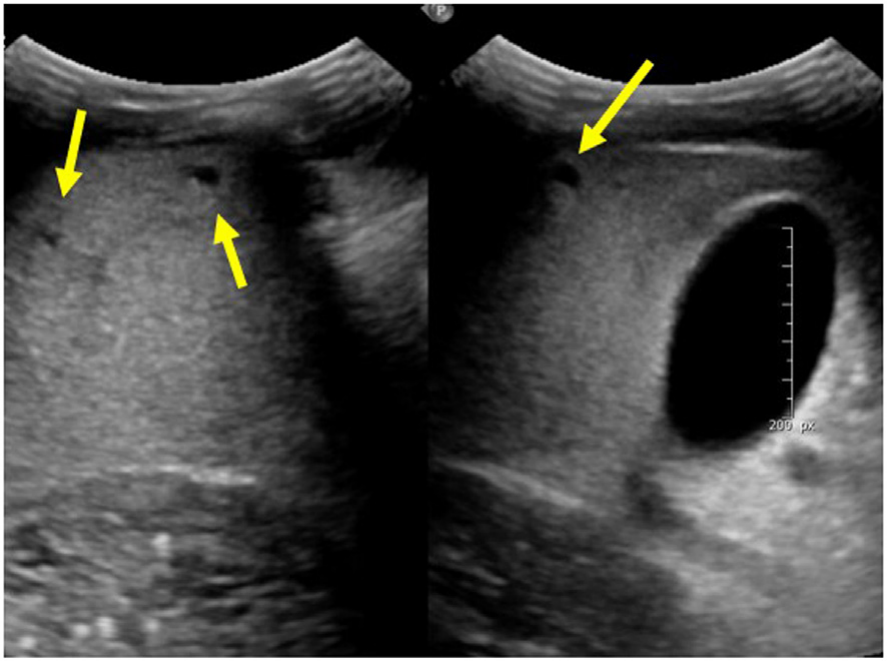
**Ultrasound images of the liver showing multiple hepatic cysts in a patient with autosomal recessive polycystic kidney disease**.

Autosomal recessive polycystic kidney disease-related liver disease can be diagnosed on a variety of imaging modalities including US, computed tomography (CT), and magnetic resonance cholangiopancreatography (MRCP). Commonly, US is the initial imaging modality due to the low cost and lack of radiation. Ectatic biliary ducts and biliary cysts can be seen on US; however, it can sometimes be difficult to distinguish between isolated hepatic cysts and cysts that are in communication with the biliary tree. Cross-sectional imaging allows for the most precise imaging of the intrahepatic ducts and can be helpful when evaluating for other biliary pathology such as choledochal cysts, biliary cysts, hepatic cysts, and Caroli disease (7). This is an important distinction to make because isolated hepatic cysts have a lower risk of infection and cholangitis compared to ectatic biliary ducts. MRCP has the advantage of no radiation or contrast necessary when compared with CT and may provide better imaging of the biliary system; however, in young children, it may require general anesthesia (7). Figure 1 shows multiple hepatic cysts seen on US in a patient with ARPKD.

There are no specific guidelines for imaging in ARPKD-related liver disease. Some centers have recommended at least one cross-sectional image to confirm diagnosis (5). It is unknown if there is any benefit to routine imaging of patients with ARPKD. Imaging studies typically do not change management as long as patients are clinically stable. New symptoms such as fever, abdominal pain, or jaundice, may warrant repeat imaging to determine if there is any progression of disease or new findings, but it is unknown if routine screening can lead to earlier diagnosis of complications.

## Management of CHF in ARPKD

There is a wide range in the severity of clinical manifestations of ARPKD, and management of hepatic disease is based on controlling specific symptoms, largely portal hypertension and cholangitis. Portal hypertension is difficult to directly measure, so indirect measures, such as spleen size or platelet count, are often used. Several studies have described portal hypertension in 36–65% of patients with ARPKD (9–11). A large, prospective study of patients with ARPKD by Gunay-Aygum et al. found splenomegaly based on MRI in 65% of patients and a statistically significant difference in platelet count between those patients with splenomegaly and those without, consistent with portal hypertension (9). A retrospective single center study by Capisonda found splenomegaly in 36% of patients over the age of 1 year (10). Systematic review found portal hypertension in 47% of patients with ARPKD (11). Chronic portal hypertension can manifest clinically with esophageal varices that have the potential for rupture. Several studies have shown an esophageal variceal rupture in 5–15% of patients with ARPKD, as shown in Table 1 (9, 10, 12). Determining the number of patients with portal hypertension and esophageal varices is difficult because there are no current guidelines for routine screening with endoscopy. Acute esophageal variceal bleeds are serious complications requiring prompt treatment, which includes endoscopy with banding or sclerotherapy. In children, given the relative low mortality associated with esophageal bleeding, primary prophylaxis (i.e., endoscopy with intervention such as variceal banding or sclerotherapy before first episode of bleeding) is not recommended in children with liver disease. Instead, intervention for esophageal varies and subsequent surveillance scope is only performed after first episode of esophageal variceal bleed. In children with recurrent variceal bleed despite routine intervention, surgical shunt to relieve portal pressure may be considered. Portosystemic shunting works to decrease portal pressures by allowing blood to bypass the fibrotic liver. There is a risk of hepatic encephalopathy with portosystemic shunting, and this may be an important consideration in patients with concomitant kidney disease and uremia. Additionally, in those with compensated cirrhosis, portosystemic shunting can also precipitate in hepatic decompensation (13). Unfortunately, the mortality associated with esophageal variceal bleeding is much higher in adults (20% in those with cirrhosis) and, therefore, when children with known CHF are transitioned to adult providers, care should be made to include an adult gastroenterologist on their team who may consider changes in management depending on the patient’s degree of portal hypertension and overall health (14). Hepatopulmonary syndrome (HPS) is another complication of cirrhosis due to intrapulmonary vasodilation; however, it is not unique to patients with ARPKD. Screening for HPS with pulse oximetry should be done for all patients with significant portal hypertension; however, gold standard testing is done with contrast enhanced echocardiography (15, 16).

**Table 1:** **Selected studies describing portal hypertension and esophageal varices in autosomal recessive polycystic kidney disease**.

	Study type	*n*	Definition of portal hypertension	Portal hypertension	Underwent endoscopy	Varices on endoscopy	Number of patients with variceal bleed
Capisonda et al.	Retrospective	27	Patent portal vein, hepatofugal portal flow, splenomegaly, and the presence of varices	10/27 (37%)	Unknown	Unknown	3/27 (11%)
Gunay-Aygum et al.	Prospective	72	Spleen length/height ratio	47/72 (65%)	31/72 (43%)	24/31 (77%)	4/72 (5%)
Luoto et al.	Retrospective	27	Splenomegaly plus thrombocytopenia (platelet count <150) OR esophageal varices	13/27 (48%)	16/27 (59%)	8/27 (30%)	4/27 (15%)
Srinath et al.	Systematic Review	788	Splenomegaly, esophageal varices, or hypersplenism	286/639 (45%)	Unknown	92/315 (29%)	38/315 (12%)

Cholangitis due to bile stasis in large, cystic bile ducts is another important consideration in the management of patients with ARPKD. Patients with known ARPKD or CHF who present with abdominal pain or fevers should be evaluated for evidence of cholangitis, cholelithiasis, and sepsis with hepatic function panel, GGT, blood cultures, CBC, and imaging such as US. Broad spectrum antibiotics should be considered as well. Rare, acute episodes of cholangitis can be treated with intermittent courses of antibiotics, but if these episodes become recurrent or chronic, suppressive antibiotics may be required. Recurrence of chronic infections can be difficult to manage because without good bile flow, antibiotics alone may not be able to treat the infection completely. Ursodeoxycholic acid is a secondary bile acid commonly used to increase bile flow, but there is limited evidence showing that it can help treat and prevent hepatolithiasis or cholangitis in patient with Caroli’s syndrome (17).

Ultimately, if the complications of portal hypertension or recurrent cholangitis are not able to be managed medically, liver transplantation can be a consideration. Liver transplantation is curative of hepatic manifestations of ARPKD; however, there are significant risks associated with transplantation and lifelong immunosuppression. If there is significant renal disease in addition to liver disease, a combined liver and kidney transplant can be performed. In general, patients with CHF who undergo liver transplant or combined liver/kidney transplant have excellent outcomes with a 1-year patient survival of 86% after liver transplant and 100% after combined liver kidney transplant in one study, though it should be noted that there were only eight patients who received combined liver/kidney transplants in this study (18). A review of the United Network for Organ Sharing database demonstrated that children with fibrocystic liver–kidney disease are more likely to receive kidney than liver transplants during childhood and the risk of subsequently needing an organ transplant in an alternate organ (i.e., requiring liver transplant after isolated kidney transplant and vice versa) is low. Furthermore, mortality for simultaneous kidney and liver transplant is comparable to single organ transplants (19). There is immunologic advantage of simultaneous kidney liver transplant as patients are less likely to have rejection in simultaneous transplants compared to kidney transplant alone (20). Therefore, when a patient is undergoing an evaluation for isolated kidney transplant, there should be careful consideration of combined simultaneous kidney and liver transplant, particularly in those with history of cholangitis who may be at risk for recurrent cholangitis after immune suppression.

## Conclusion

Hepatic manifestations of ARPKD are due to embryologic ductal plate malformation that manifests as large, cystic bile ducts, and CHF. Clinically, patients with liver disease due to ARKD can present with recurrent cholangitis or portal hypertension, as manifested by ascites, variceal bleeding, splenomegaly, and thrombocytopenia. Diagnosis of ARPKD is often made by genetic testing, but hepatic manifestations are readily seen on multiple imaging modalities including US, MRI, and CT. Management of liver disease is aimed at treating specific complications including antibiotics for cholangitis or endoscopic therapy for variceal bleeding. Liver transplantation is curative of liver disease in ARPKD, but reserved for patients whose symptoms cannot be managed medically.

## Author Contributions

AW, AK, and JW contributed to the conception of the work and drafting. JW provided editing and final approval of the manuscript. All authors discussed the content at all stages of the manuscript.

## Conflict of Interest Statement

JW receives research support from Gilead Sciences, Inc., Bristol-Myers Squibb, AbbVie, Inc., and Roche that are not relevant to the current manuscript. The other authors declare no conflict of interest.
